# Concerted Action Is Needed to Tackle Liver Fluke Infections in Asia

**DOI:** 10.1371/journal.pntd.0000232

**Published:** 2008-05-28

**Authors:** Banchob Sripa

**Affiliations:** 1 Asian Liver Fluke Network, Khon Kaen, Thailand; 2 Tropical Disease Research Laboratory, Department of Pathology, Faculty of Medicine, Khon Kaen University, Khon Kaen, Thailand; 3 Liver Fluke and Cholangiocarcinoma Research Center, Khon Kaen University, Khon Kaen, Thailand; Queensland Institute of Medical Research, Australia

Human liver fluke infection caused by *Clonorchis sinensis*, *Opisthorchis viverrini*, and *O. felineus* remains a major public health problem, affecting the poor in the poorest regions of Asia. An estimated 45 million people are infected and more than 600 million are at risk of these infections [1],[2]. This Viewpoint highlights the distribution of the infection, clinical features, neglect of liver fluke infection, and how the global health community can help to eradicate the infection in Asia.

## Distribution of the Infection

Most of those infected with liver fluke live in Asia. *C. sinensis* is endemic in south China, northern Vietnam, Taiwan, and Korea, while *O. viverrini* is found mainly in Thailand, Lao People's Democratic Republic (PDR), Cambodia, and central Vietnam. In Northeast Thailand and Laos, despite widespread administration of the anthelmintic drug praziquantel, the prevalence of *O. viverrini* approaches 85% in endemic areas in Lao PDR [3]. A recent survey in the Chi River basin in northeast Thailand found a prevalence of *O. viverrini* of up to 78% in certain villages (B. Sripa, T. Laha, J. Bethony, P. J. Brindley, et al., unpublished data). An estimated 6 million people in Thailand are infected with *Opisthorchis* [4].

## Clinical Features

**Figure 1:**
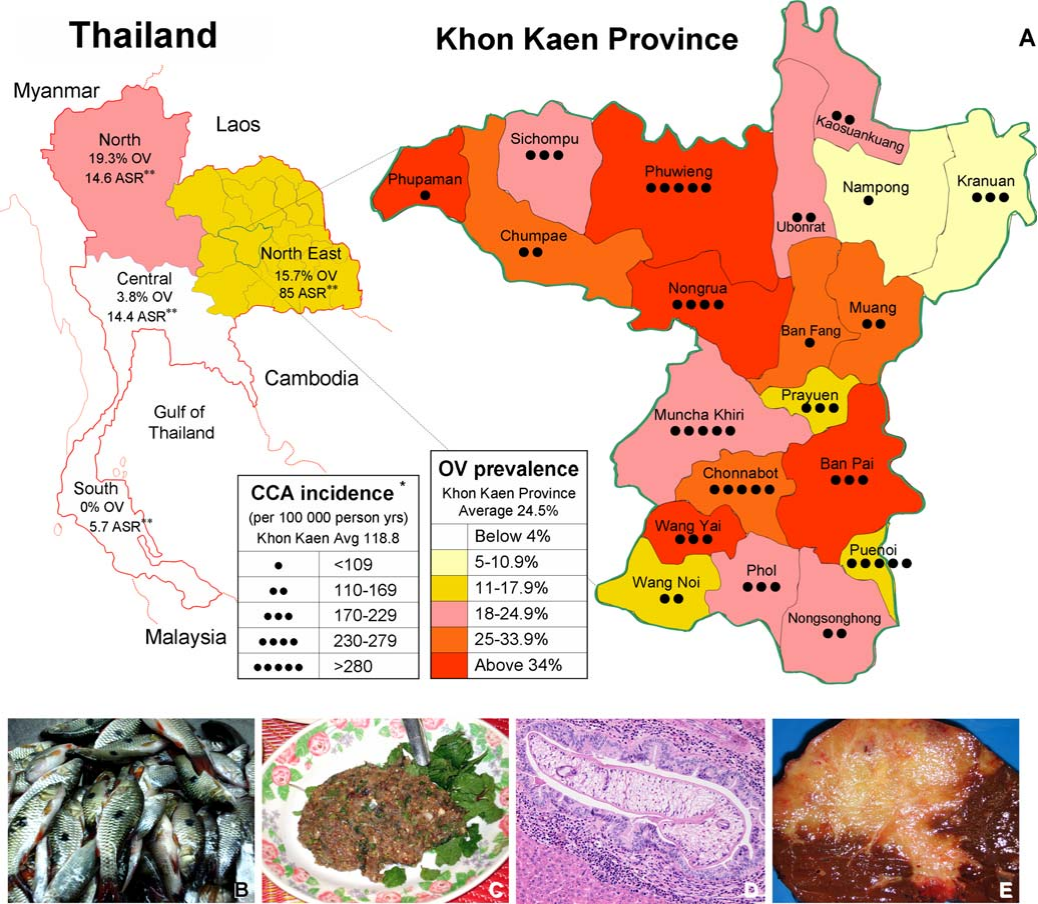
Incidence of CCA and *O. viverrini* in Thailand from 1990 to 2001. (A) Increasing intensity of red represents increasing prevalence of *O. viverrini*, while increasing number of dots represents increasing cancer rates. In general, higher *O. viverrini* prevalence correlates with a higher CCA burden, although sporadic anthelmintic therapy has influenced this relationship. It should be noted that even one spot represents significant cancer rates anywhere else in the world. *Truncated age-standardized incidence from 35 to 64 years. **Age-standardized incidence of CCA throughout registered regions [6]. (B–E) Cyrinoid fishes that represent the intermediate host of the *O. viverrini* parasite (B); a dish of *koi-pla* (minced fish and condiments), which is thought to represent a common source of infectious metaceraciae of *O. viverrini* (C); photomicrograph of an adult *O. viverrini* worm in bile ducts of experimentally infected hamster (D); photograph of cholangiocarcinoma in human liver from a patient from Khon Kaen, Thailand (E). (Modified from Sripa et al. [5].)

Liver fluke infections are associated with several hepatobiliary diseases, including cholangitis, cholecystitis, gallstones, hepatomegaly, and cholangiocarcinoma (CCA), the primary liver cancer that arises from biliary epithelial cells [5]. *Opisthorchis* is one of two helminth parasites (*O. viverrini* and *Schistosoma hematobium*) that are carcinogenic to humans, as reported by the World Health Organization and the International Agency for Research on Cancer [6]. In Thailand, CCA is the most prevalent of the fatal neoplasias, and liver and bile duct cancer ranks at number five in the list of diseases in Thailand that cause the highest number of disability adjusted life years (DALYs) [7]. CCA is responsible for about 15%–25% of liver cancers in the United States but represents 86.5% of all cancers in Thailand's Khon Kaen region, the highest incidence in the world (Figure 1) [5].

## The Neglect of Liver Fluke Infections

Despite the high prevalence of liver flukes in Asia, and the associated hepatobiliary disease, these infections are relatively neglected, not only by national governments but also by international health organizations and granting agencies. Because of the rapid economic growth during the past decade in Asia, an epidemiological transition has occurred—there has been an increase in “Western” life styles (such as adopting Western diets) and a rise in the so-called chronic diseases of affluence, particularly in the middle class population. For example, in Thailand, the top four causes of death in the year 2004 were heart disease, diabetes, hypertension, and cancer [7]. The Thailand National Health Strategic Plan 10 (2007–2011) therefore mainly focuses on these diseases and emerging infections, i.e. avian influenza, HIV/AIDS, influenza, and tuberculosis (TB) [8]. Other infectious diseases with high morbidity/mortality such as malaria, dengue, leptospirosis, filariasis, and leprosy are always on the list of priorities of the Ministry of Public Health [9]. However, asymptomatic parasitic infections, including those caused by liver fluke, that affect the poor and poorest people (i.e., the majority of the population) have received less attention. A major Thai campaign to eliminate liver fluke that was operational a decade ago has now disappeared, even though the infection is still endemic in northeastern and northern parts of Thailand [4]. Similar situations have been described in other liver fluke endemic countries of Asia [10].

At the regional and global level, liver flukes are also neglected. For example, they are not listed in the current disease portfolio of the Special Programme for Research and Training in Tropical Diseases [11]. The WHO Control of Neglected Tropical Diseases (NTDs) project currently lists 14 targeted tropical diseases (Box 1), but liver flukes are excluded [12]. Moreover, liver flukes are not included in the WHO's strategic plan, *Global Plan to Combat Neglected Tropical Diseases 2008–2015* [13]. In Asia, the WHO Regional Offices for Southeast Asia (SEARO) and the Western Pacific (WPRO) focus on major communicable diseases in the region such as HIV/AIDS, TB, influenza, malaria, and dengue [10],[14]. SEARO clearly stated plans to eradicate and eliminate four neglected tropical diseases, including leprosy, lymphatic filariasis, kala-azar, and yaw in the strategic plan for 2008–2009, but not liver flukes [14].

**Box 1. The 14 Target Diseases of the WHO's Control of Neglected Tropical Diseases Department (from [12])**

- Buruli ulcer
- Chagas disease
- Cholera/epidemic diarrhoeal diseases
- Dengue/dengue haemorrhagic fever
- Dracunculiasis (guinea worm)
- Endemic treponematoses (yaws, pinta, endemic syphilis)
- Human African trypanosomiasis (sleeping sickness)
- Leishmaniasis
- Leprosy
- Lymphatic filariasis
- Onchocerciasis
- Schistosomiasis
- Soil-transmitted helminthiasis
- Trachoma

Because of the neglect by health policymakers at national, regional, and global levels, liver flukes are not recognized as targeted diseases for grant funding. During the past few years, there has been increased interest shown by several agencies in supporting the control of neglected tropical diseases. For example, in 2006, the United States Agency for International Development (USAID) awarded RTI International $100 million for a neglected tropical diseases control project, with the goal of delivering integrated treatments to 40 million people in Africa over 5 years [15]. The Bill & Melinda Gates Foundation has launched global health research grants with a total budget of $46.7 million to fight against NTDs [16]. However, these two major granting agencies appear only focus on those NTDs that are listed by WHO [12]. Meanwhile, important national research in poor Asian countries that is crucial to the control of liver fluke infection remains sidelined by granting agencies.

Those suffering from liver fluke infection, and those working to eradicate it, have very little voice. In an effort to remedy this situation, researchers drawn from Asian countries where liver fluke is endemic, including Thailand, Lao PDR, Vietnam, Cambodia, China, and Korea, agreed to set up the Liver Fluke Network. The formal establishment of this network took place on 5–10 September 2007 at the 7th Regional Network on Asian Schistosomiasis and Other Helminth Zoonoses (RNAS^+^) Workshop and the First International Symposium on Geospatial Health held in Lijiang, Yunnan Province, People's Republic of China. The network's shared concern, which brings us together, is that the liver fluke infections are neglected diseases in the official list of neglected tropical diseases. The aim of the network is to exchange expertise, collaborate, and cooperate in liver fluke research to fight against these infections in Asia (the specific aims are shown in Box 2). The network is promoting research not only on the epidemiology of the disease, but also on basic science studies on liver fluke biology, molecular biology, pathobiology, immunology, diagnosis, and vector biology. Details of our proposed strategic plan and activities can be viewed at http://www.liverfluke.net/.

**Box 2. The Specific Aims of the Liver Fluke Network (www.liverfluke.net/)**

1. To promote interaction between scientists who are working in liver fluke research in Asia.
2. To facilitate research capacity building and technical support to Liver Fluke Network members through dissemination of information on training opportunities in relation to research on the control of the liver flukes.
3. To identify and disseminate information on funding opportunities for liver fluke research.
4. To share plans for new liver fluke research areas and exploring potential projects for international collaboration.
5. To facilitate the translation of basic research findings into practical applications, particularly those relevant to the prevention and control of liver fluke infections and related hepatobiliary diseases.

However, the Liver Fluke Network has only just started and we have no definite funding support. We therefore seek collaboration and affiliation with health organizations, institutions, and foundations to move our plan forward. Because of our budget constraints, we plan to have the first meeting at the 17th International Congress for Tropical Medicine and Malaria in Jeju Island, Korea, on September 29–October 3, 2008 (http://www.ictm17.org/), where we will organize a symposium on liver fluke diseases. We, researchers from endemic regions, hope that the global health community, especially health organizations, governmental and private foundations, and granting agencies, will consider supporting our activities. It is time to put liver fluke infection firmly on the global health agenda.

## Supporting Information

Alternative Language Abstract S1 Summary of the article translated into Thai by Banchob Sripa.
(0.03 MB DOC)

## References

[1] Lun ZR, Gasser RB, Lai DH, Li AX, Zhu XQ (2005). Clonorchiasis: a key foodborne zoonosis in China.. Lancet Infect Dis.

[2] Keiser J, Utzinger J (2005). Emerging foodborne trematodiasis.. Emerg Infect Dis.

[3] Sayasone S, Odermatt P, Phoumindr N, Vongsaravane X, Sensombath V (2007). Epidemiology of *Opisthorchis viverrini* in a rural district of southern Lao PDR.. Trans R Soc Trop Med Hyg.

[4] Jongsuksuntigul P, Imsomboon T (2003). Opisthorchiasis control in Thailand.. Acta Trop.

[5] Sripa B, Kaewkes S, Sithithaworn P, Mairiang E, Laha T (2007). Liver fluke induces cholangiocarcinoma.. PLoS Med.

[6] IARC (1994). Schistosomes, liver flukes and *Helicobacter pylori*. IARC Working Group on the Evaluation of Carcinogenic Risks to Humans. Lyon, 7–14 June 1994.. IARC Monogr Eval Carcinog Risks Hum.

[7] International Health Policy Program, Ministry of Public Health (2007). Burden of diseases and injuries in Thailand. Medium-term report [in Thai].. http://www.ihpp.thaigov.net/bod/index.html.

[8] Ministry of Public Health (2007). The National Health Development Plan 2007–2051 (under the Ninth National Economic and Social Development Plan) [in Thai].. http://www.moph.go.th/other/inform/10/index.htm.

[9] Bureau of Policy and Strategy, Ministry of Public Health (2005). Thailand health profile 2001–2004.. http://www.moph.go.th/ops/health_48/index_eng.htm.

[10] World Health Organization Regional Office for the Western Pacific (2008). Programmes and special initiatives.. http://www.wpro.who.int/sites/.

[11] World Health Organization Special Programme for Research and Training in Tropical Diseases [TDR] (2008). TDR diseases.. http://www.who.int/tdr/diseases/default.htm.

[12] World Health Organization (2006). Neglected tropical diseases, hidden successes, emerging opportunities.. http://whqlibdoc.who.int/hq/2006/WHO_CDS_NTD_2006.2_eng.pdf.

[13] World Health Organization (2007). Global plan to combat neglected tropical diseases 2008–2015.. http://whqlibdoc.who.int/hq/2007/WHO_CDS_NTD_2007.3_eng.pdf.

[14] World Health Organization Regional Offices for Southeast Asia (2007). Department of Communicable Diseases: Profile and vision, revised 2007.. http://www.searo.who.int/en/Section10_11520.htm.

[15] The United States Agency for International Development (USAID) (2006). Control of neglected tropical diseases.. http://www.usaid.gov/our_work/global_health/id/ntd_brief.pdf.

[16] Bill & Melinda Gates Foundation (2006). Leading global health organizations receive $46.7 million from Gates Foundation to integrate programs fighting neglected tropical diseases [press release].. http://www.gatesfoundation.org/GlobalHealth/Announcements/Announce-061219.htm.

