# Supplementary material for: Concerted Action Is Needed to Tackle Liver Fluke Infections in Asia
Source: PLoS Negl Trop Dis. 2008 May 28;2(5):e232. doi: 10.1371/journal.pntd.0000232 (PMC2386259; doi:10.1371/journal.pntd.0000232)
Supplement: Alternative Language Abstract S1 — Summary of the article translated into Thai by Banchob Sripa. (0.03 MB DOC) [file pntd.0000232.s001.doc]

**Manuscript number 08-PNTD-VP-0031R1**

**Author summary in Thai language**

Concerted Action Is Needed to Tackle Liver Fluke Infections in Asia

ไม่มีความสัมพันธ์ใดๆ ระหว่างการติดเชื้อก่อโรคที่เป็นสัตว์หลายเซลล์กับการเกิดมะเร็งมากกว่าความสัมพันธ์ระหว่างการติดพยาธิใบไม้ตับชนิด *Opisthorchis viverrini*  กับการเกิดมะเร็งท่อน้ำดี ในประเทศไทยถึงแม้จะมีการรณรงค์กำจัดพยาธิด้วยยาพราซิควัลเทลมานานแต่ก็ยังมีความชุกของการติดพยาธิใบไม้ตับชนิดนี้สูงถึง 70 เปอร์เซ็นต์ในบางพื้นที่ นอกจากนี้ประเทศไทยยังมีอุบัติการณ์ของมะเร็งท่อน้ำดีสูงในพื้นที่ที่มีการระบาดของพยาธิสูงด้วย โรคติดเชื้อพยาธิใบไม้ตับก็เหมือนกับโรคติดพยาธิใบไม้ที่ติดจากการรับประทานอาหารชนิดอื่นๆ ที่เป็นโรคที่ถูกละเลย บทความนี้จึงพยายามที่จะชี้นำให้ประชาคมโลกได้ตระหนักถึงปัญหาของโรคในกลุ่มนี้ และนักวิจัยในภูมิภาคเอเชียจึงได้ร่วมกันก่อตั้งเครือข่ายวิจัยพยาธิใบไม้ตับแห่งเอเชียขึ้นมาเพื่อศึกษาวิจัยในทุกแง่มุมของโรคนี้
